# Auto-Configuration Protocols in Mobile *Ad Hoc* Networks

**DOI:** 10.3390/s110403652

**Published:** 2011-03-25

**Authors:** Luis Javier García Villalba, Julián García Matesanz, Ana Lucila Sandoval Orozco, José Duván Márquez Díaz

**Affiliations:** 1 Grupo de Análisis, Seguridad y Sistemas (GASS), Departamento de Ingeniería del Software e Inteligencia Artificial (DISIA), Facultad de Informática, Despacho 431, Universidad Complutense de Madrid (UCM), Calle Profesor José García Santesmases s/n, Ciudad Universitaria, 28040 Madrid, Spain; E-Mail: asandoval@fdi.ucm.es (A.L.S.O.); 2 Grupo de Análisis, Seguridad y Sistemas (GASS), Sección Departamental de Sistemas Informáticos y Computación—Lenguajes y Sistemas Informáticos y Ciencias de la Computación e Inteligencia Artificial, Facultad de Ciencias Matemáticas, Despacho 310-F, Universidad Complutense de Madrid (UCM), Plaza de Ciencias, 3, Ciudad Universitaria, 28040 Madrid, Spain; E-Mail: julian@sip.ucm.es (J.G.M.); 3 Departamento de Ingeniería de Sistemas, Universidad del Norte, Km. 5 Autopista a Puerto Colombia, Barranquilla, Colombia; E-Mails: alsandoval@uninorte.edu.co (A.L.S.O.); jmarquez@uninorte.edu.co (J.D.M.D.)

**Keywords:** auto-configuration protocol, Mobile *Ad Hoc* Network (MANET), IP address assignment, distributed dynamic host configuration protocol, D2HCP, routing protocol

## Abstract

The TCP/IP protocol allows the different nodes in a network to communicate by associating a different IP address to each node. In wired or wireless networks with infrastructure, we have a server or node acting as such which correctly assigns IP addresses, but in mobile *ad hoc* networks there is no such centralized entity capable of carrying out this function. Therefore, a protocol is needed to perform the network configuration automatically and in a dynamic way, which will use all nodes in the network (or part thereof) as if they were servers that manage IP addresses. This article reviews the major proposed auto-configuration protocols for mobile *ad hoc* networks, with particular emphasis on one of the most recent: D2HCP. This work also includes a comparison of auto-configuration protocols for mobile *ad hoc* networks by specifying the most relevant metrics, such as a guarantee of uniqueness, overhead, latency, dependency on the routing protocol and uniformity.

## 1. Introduction

A *Mobile Ad hoc NETwork* (MANET) is a set of mobile nodes which communicate between themselves through wireless links. In contrast with conventional networks, a MANET does not need any previous infrastructure, since nodes rely on each other to operate themselves, forming what is called multi-hop communication.

Such networks have more problems and disadvantages than a conventional network. The topology of mobile networks may change quickly and in an unpredictable way. Moreover, variations in the capacity of nodes and links, frequent transmission errors and a lack of security may occur. Finally, the limited resources of the nodes must be taken into account given that an *ad hoc* network will normally be formed by battery operated devices.

To communicate with each other [1] the *ad hoc* nodes need to configure their interfaces with local addresses which are valid within the *ad hoc* network. The *ad hoc* nodes may also need to set global routing addresses to communicate with other devices on the Internet. From the perspective of the IP layer, an *ad hoc* network presents itself as a multi-hop level 3 network constituted by a collection of links.

This paper is organized into six sections, including this introduction. Section 2 tackles the problematic implications of the design of auto configuration protocols for mobile *ad hoc* networks. Section 3 discusses the inapplicability of the standard solutions. In Section 4, a classification of auto-configuration protocols for mobile *ad hoc* networks is carried out, with the most representative being mentioned, and special emphasis being made on a protocol proposed by the author: D2HCP. Then, in Section 5, a comparative study of the above-mentioned protocols is performed. Finally, Section 6 of the paper draws together the conclusions of this piece of work.

## 2. Auto-Configuration in Mobile *Ad Hoc* Network

The nodes of a network need some mechanism to interchange messages with each other. The TCP/IP protocol allows the different nodes from the network to communicate by associating a distinct IP address to each node of the same network. In wired or wireless networks with an infrastructure, there is a server or node which correctly assigns these IP addresses.

Mobile *ad hoc* networks, on the other hand, do not have such a centralized entity able to carry out this function. Therefore, some protocol that performs the network configuration in a dynamic and automatic way is necessary, which will utilize all the nodes of the network (or only part of them) as if they were servers which manage IP addresses.

Due to the dynamic topology of mobile *ad hoc* networks (constant movement of the nodes that can join and leave the network frequently and even simultaneously), auto-configuration protocols are faced with various problems in guaranteeing the uniqueness of IP addresses and in allowing network partitioning and merging.

To guarantee the correct functioning of the network, the protocols strive to achieve the following objectives [2–4]:

- *Assign unique IP addresses*: Ensure that two or more nodes do not obtain the same IP address.
- *Function correctly*: An IP address is only associated with a node for the time that it is kept in the network. When a node leaves the network, its IP address should then became available for association to another node.
- *Fix the problems derived from the loss of messages*: In case of any node failure or if message loss occurs, the protocol should operate quick enough to prevent two or more nodes from having the same IP address.
- *Allow multi-hop routing*: A node will not be configured with an IP address if there aren’t any available in the whole network. Thus, if any node of the network has a free IP address, it has to associate itself with the node which is requesting an IP address, even though it is at two-hops of distance or more.
- *Minimize the additional packet traffic in the network*: The protocol must minimize the number of packets exchanged among the nodes in the auto-configuration process. In other words, control packets traffic must cause as little harm as possible to the data packet traffic, given that in the extreme case, the network performance would decrease.
- *Verify the existence of competing petitions for an IP address:* When two nodes request an IP address at the same time, the protocol must carry out the pertinent treatment so that the same IP address is not given to two nodes.
- *Be flexible to partitioning and merging of the mobile ad hoc network*: The protocol must be able to achieve the union of two different mobile *ad hoc* networks as well as the possible partitioning into two networks.
- *Conduct synchronization*: The protocol must adapt itself to the rapid changes of the wireless network topology due to the frequent mobility of the nodes. The synchronization is carried out periodically to ensure the configuration of the network is as up to date as possible.

## 3. Applicability of Standard Solutions

The applicability of the standard protocols is insufficient for MANET [5]. Two of these protocols are presented next.

### 3.1. SLAAC/NDP

*StateLess Address AutoConfiguration* (SLAAC) [6] is a standard that allows the automatic auto-configuration of an IPv6 address without the need of a router node. For this, it needs help from the protocol *Neighbour Discovery Protocol* (NDP) [7], a standard to transmit the messages and to discover its neighbours. A node automatically creates an IPv6 address joining its *host* identifier (it is usually the MAC address) with a local well-known prefix and undergoes a DAD process by *broadcasting* NDP messages to the neighbours.

If the IPv6 address is not unique, the auto-configuration process will stop and it will be necessary to do it manually. If on the contrary the address is unique, it will have to ask for the network prefix through NDP messages and, then, if its IPv6 address is effectively unique, it will verify with DAD again.

The applicability of this protocol in mobile *ad hoc* networks is limited because it uses the NDP protocol to send the messages and NDP assumes that all the nodes are connected to each other in the network. Consequently, it only supports one-hop transmission, whereas mobile *ad hoc* networks are most frequently multi-hop, thus not reaching the majority of the nodes to carry out the DAD processes and not being able, therefore, to ensure that the obtained IPv6 address is unique.

### 3.2. DHCP-PD

*Dynamic Host Configuration Protocol – Prefix Delegation* (DHCP-PD) [8] is an option derived from DHCPv6 [9] which provides a mechanism for the delegation of the IPv6 address prefixes and lets one of these be assigned automatically. For this, if a node wishes to get an IPv6 address, it sends a DHCP message with the activated *prefix delegation* option to obtain a prefix from a DHCP server in the network.

Its applicability is limited in mobile *ad hoc* networks because it is based on DHCP, so it assumes that all the nodes can connect to a DHCP server, whether directly or through several hops, and due to the MANET topology, the direct connection to DHCP server is not usually frequent with the consequence that the connection through several hops can make the server unreachable.

## 4. Classification of Auto-Configuration Protocols

The auto-configuration protocols may be classified according to address management:

- *Stateful*: The nodes know the network state, *i.e.*, they keep tables with the IP addresses of the nodes.
- *Stateless*: The IP address of a node is managed by itself. Generally they create a random address and perform a process of duplicated address detection steps to verify their uniqueness.
- *Hybrid Protocols*: They mix mechanisms from the previous ones to improve the scalability and reliability of the auto-configuration. Their algorithms have a high level of complexity.

### 4.1. Stateful Protocols

#### 4.1.1. MANETConf

MANETConf [10], which is an improvement of [11], is based on existence of a common distributed table so that all the nodes are able to assign IP addresses. When a node wants to join the network, it sends broadcast messages to other nodes and the first one which replies to the message, chooses it as an initiator node and it can supply an IP address. The initiator node chooses one of the free IP addresses located in the network and before assigning it, asks for permission from the rest of the nodes, because it is possible that another node may have also chosen this address or the tables might not be totally synchronized due to message delays. If the answer from the nodes is positive, it assigns the IP address to the joining node and it communicates this action by *broadcasting* so that the rest of the nodes keep their tables updated. If there is a node which does not reply, it is put in direct contact with it (*unicast*) to get an answer. If it still cannot get it, it will assume that the node has left the network, and will communicate this to the rest of the nodes in the network in order to keep the tables updated.

#### 4.1.2. DAAP

Dynamic Address Allocation Protocol (DAAP) [12] is based on the concept of address assignment by a leader. The leader functionality is shared among all network nodes. When a new node joins the network, it becomes the leader until the next node joins. The leader maintains the highest IP address within the *ad hoc* network and a unique identifier is associated with the network.

Each node stores the highest IP address, which is that of the leader, and periodically sends HELLO messages to its neighbours. These HELLO messages include the network identifier so that any merging and partitioning can be detected. When a node receives a HELLO message with a different network ID, merging is detected, if a node does not receive the message that contains the current network ID, then after a timeout, a partition is detected.

#### 4.1.3. Moshin and Prakash’s Protocol

The Moshin and Prakash proactive scheme [13] tries to fix the problem of IP address assignment by binary division of free address blocks. These divisions are done to the power of 2. Thus, nodes can exist with any block. Moreover, each node of the network has a table with the state of all the others in the network; that is to say, it knows the free address blocks of other nodes, as well as the IP address of the network interface from each node.

The address assignment process can come from any node. Following this idea, a non-configured node (client node) sends a *broadcast* message of the REQUEST type so that a node in the network configures this client node. As it is a diffusion message, it is possible to have several replies, but it will choose the node that replies first (REPLAY), and this will act as the server node. If this node has several blocks, it will give one to the client node, and will choose the first IP address from the block as its own. Otherwise, if the node has a block, then it will divide into two similar parts and will give one half to the client node, and keep the other one for itself.

If we consider a case in which there aren’t any blocks available, some solutions are proposed based on the idea that the server searches its surrounding neighbours to see whether any block is available. In the case where no free addresses are found, it will try to get a block by using the neighbors of a higher level hop, and so on. Another solution consists of looking for the node that has the greater free range address, to deliver half of this block to the node which is joining the network.

The departure of the nodes can take place in an abrupt or smooth way, and for each case there is a scheme to follow. If the departure is abrupt, the node that acted as the server in its configuration will see in its routing table that the block of node that left the network is not there and it will add the block to its free address block. In the case of easy departure, the node, which leaves the network, warns it’s neighbours of its intention to leave and the neighbour node then seeks the node which configured the client so that it gets its block.

The greatest advantages of this protocol is that it works well for merging and partitioning of networks, since it fixes the duplicated addresses problem which occurs in these cases. Each node which initiates the network generates a random number called *PartitionID* which will be a network identification number. In this manner, when a partitioning or merging of the network occurs, the first node which leaves from original network will create another *PartitionID*. At the time the two networks with different PartitionID are joined; firstly the consistency of its IP address will be checked. This process consists of verifying the existence of two nodes with the same address. In case affirmative, a change of the node belonging to the network with less free IP address range is produced. The new IP address will belong to the highest network range with the biggest free address number. The major drawback of this protocol is that the synchronization depends on the existence of a reliable *broadcast* and such a thing does not exist in a distributed mobile environment, thus one can question the robustness of this protocol.

#### 4.1.4. Thoppian and Prakash’s Protocol

An improvement of the previous scheme (particularly in terms of synchronization) can be found in [14], where Thoppian and Prakash propose a dynamic address assignment based on a so-called *buddy system* that manages mobility of nodes during address assignment, message loss, network partitioning and merging. However, the IP address allocation can generate a high overhead of control messages while it does a global search and the address recovery (to avoid missing addresses) requires diffusion messages by a *flooding* process. In addition, union and partition may incur in high overhead because of the global nature of this protocol.

#### 4.1.5. EMAP

*Extensible Manet Auto-configuration Protocol* (EMAP) [15] is an auto-configuration protocol based on the idea of a protocol of REQUEST/REPLAY messages. The main advantage of this protocol is the possibility of doing it extensibly, *i.e.*, it can include new functionalities in the future that are analyzed in a theoretical way, such as *Domain Name Server* (DNS).

This protocol also considers the possibility of exterior communications to the mobile *ad hoc* network via Internet. The route discovery mechanism among nodes is similar to the *Ad Hoc On-Demand Distance Vector* (AODV) [16] protocol.

The main idea of this auto-configuration protocol consists in having three different addresses for a non-configured node that wants to join the created network. These are three addresses for the interior communications: *temporary address*, *tentative address* and *mobile* ad hoc *network local address*.

When a node wishes join the network, it randomly generates two valid IP addresses (with network known addresses), considering them as temporary and tentative addresses. These IP addresses are encapsulated into a *Detection Adress Detection REsPonse* (DAD_REP) message to know whether it is a valid address. The node keeps waiting for a *Detection Address Detection REQuest* (DAD_REQ) message. If time runs out for this message, the node assumes that it can use its tentative address as unique, and assigns it to its network interface. If this node receives a DAD_REP message to its temporary address and this message contains the source with the tentative address that had been proposed, the node knows that this tentative address is being used and the previous process begins creating another pair of addresses again.

#### 4.1.6. Sheu *et al.*’ s Protocol

The auto-configuration protocol introduced in [17] proposes a scheme where the nodes are classified into coordinator and common nodes. The first coordinator assigned to initiate the IP address assignment is the so-called *C-root*. The coordinators manage the IP address pool and they are responsible for assigning an IP address to a node which has just joined the network. The nodes that wish to join the network will interchange *HELLO* messages to find the coordinator node nearest and to obtain a new IP address from that coordinator node. To maintain the IP address pool efficiently, the coordinator nodes are distributed in a tree topology called *C-tree* by exchanging *HELLOs.* This protocol does not consider network partition and merging.

#### 4.1.7. D2HCP

The Distributed Dynamic Host Configuration Protocol (D2HCP) [18] is an auto-configuration protocol for mobile *ad hoc* networks which derives from improvements made to the Mohsin and Prakash’s [13] protocol which guarantee the uniqueness of IP addresses used in the network. The protocol makes the nodes of a MANET work together to manage the unique and correct IP address assignment in a distributed manner. All network nodes have the same role; there is no special node type that centralizes the management.

The protocol is based on the OLSR routing protocol [19] to perform synchronization, and thus detect changes in the network. This synchronization procedure produces a null overhead in network traffic compared to that generated by the OLSR protocol. In this scheme, when a node enters the network, as well as assigning a valid IP address, a range of consecutive addresses are given. From that point on, the new node is responsible for managing the range of addresses, and can allocate part of its addresses to other nodes requesting entry into the network.

To request a new IP address, the requesting node, referred to as client, sends a *Server_Discovery* message to its neighbours. The neighbour nodes that are part of the network will answer this request by sending a *Server_Offer* message. The client node will choose one of the received responses and will send a *Server_Poll* message to the chosen neighbour node requesting an address. The node which will assign the new address will receive the server name. When the client node receives the *Server_Poll* message, it will divide its address range into two halves, and give the second half of the client. If the addresses provided by the server node were not their own, but a third node of the network, the server sends an *IP_Range_Request* message formally requesting these addresses to the node that owns the address range. The third network node sends an *IP_Range_Return* message authorizing the node that sent the *IP_Range_Request* message to assign the address block to the client node. After receiving the *Server_Poll* message, if the provided addresses were from the server node, or if *IP_Range_Return* message was necessary in the case of having had to ask for address to a third node, the node server sends an *IP_assigned* message to the client with assigned range.

Design Considerations:

- Each node has as associated information, a single block of contiguous free IP addresses for auto-configuration, which include the own IP address of the node itself.
- The responsibility of recover the IP addresses that a node makes available when it leaves the network is one that can join this free block to the right of its own. This is not possible when the block to be collected contains the lowest address of the network. In that case, the node that collects the block is one that can add to it on the left.
- By dividing the free addresses in two blocks to deliver one of them to a new node entering the network, the node that makes the client server delivers sub-block does not contain its own IP address.

### 4.2. Stateless Protocols

#### 4.2.1. Process of Duplicated Address Detections

*Duplicate Address Detection* (DAD) is a process which uses the protocols to check the uniqueness of IP addresses. This process takes a relatively long time to complete, so several solutions have been implemented to reduce it. There are three kinds of DAD processes:

- *Strong Duplicate Address Detection (SDAD)* [20]: Is the base of the Stateless protocols. It consists of a simple mechanism whereby the node chooses two IP addresses: temporary and tentative. It will only use the temporary address for the initialization while it detects if the tentative one is unique or not. The detection method consists of sending a message ICMP destined directly to this address. If it receives a response, this IP address is being used so the process will be resumed. If it does not receive a response, the message will be sent a certain number of times to make sure that the address is unique. By being a very simple mechanism, it does not ensure the uniqueness of the IP address since the process limits itself to only the phase of initialization, and it would not work for temporary disconnections or losing of the network. Moreover, when the network is long and only a few free IP addresses remain, it increases the overhead until it finds a unique IP address.
- *Weak Duplicate Address Detection (WDAD)* [21]: It establishes the idea of tolerating the duplicated address in the network for a period of time. For that, every node when it is being initiated itself will create a key that it will always send along with its IP address. When a node receives a message, it will check whether this IP address is already assigned in its table and will look whether the keys coincide, if they do not coincide, it will mark that address as invalid and actions will be taken so that they are unique (these actions are not defined in WDAD). This process has to support the identification of a node by means of a key-IP pair and depends completely on the routing protocol. It will only work with the proactive one that updates the routes constantly, but with a reactive one there will be nodes that could never detect the duplicity of IP addresses. It does not add additional overhead to the routing protocol, but on the other hand, it really adds to the overhead by sending always the key along with the IP address.
- *Passive Duplicate Address Detection (PDAD)* [22]: The idea is based on sending control information instead of detecting or solving duplicated IP addresses, every node investigates and deduces whether a duplicated address exits by events that would never happen if all the IP addresses were unique.

Three passive detections are proposed, which are necessary for correct functioning of the detection:

▪ *Sequence Numbers (PDAD-SN)*: This system is based on the idea that the routing protocols use sequence numbers in its messages to update the routes. Using these sequence numbers, and the idea that two nodes with a distance between them of two-hops do not have the same neighborhood, some conflicts are solved. Also, it has taken into consideration the possibility that these sequence numbers reach the maximum and numbers start from zero again.
▪ *Locality Principle (PDAD-LP)*: Lower power than the previous one, is based on the frequency of updating the route tables. On the basis of this frequency it can detect duplicate addresses by taking a time threshold to display the status of the route tables. It’s necessary to take into account the protocol routing used. We must consider different thresholds, since it can be the case that two messages with the same source are confused with a duplicate address, if the time is too short and, therefore, the protocol modifies the routes too fast.
▪ *Neighborhood (PDAD-NH)*: Taking into account that a node knows its neighbours, and they have sent a package of the state of its link, it differs if there is conflict or not depending on whether it is in a package, the source of this message is a neighbour, and contains the address. The advantage is that it does not add overhead to network, but it can only be used with proactive routing protocols.

#### 4.2.2. APAC

*Agent based Passive Auto-configuration* (APAC) [23] is an auto-configuration protocol based on PDAD. Its main feature is the use of certain nodes which centralize the distribution of addresses. The mechanism by which a node configures its IP address upon entering the network consists of asking if it has some node type *Address Agent* (AA) within a one hop distance. In that case, the AA node will give it an IP address.

If it does not receive a response from any AA node, the incoming node is configured to operate in AA mode, and it will be a server of addresses for the next incoming nodes. When it is configured as AA, the node randomly generates an identifier number *agentID*, in order to form a table with the addresses that it will assign to the nodes that arrive. These addresses have the form *agented* + *hostID*.

When a node is moved in the network and leaves the radio coverage of AA proportionately to its IP address, this node must ask for another address from another node AA within its new radio coverage. This is complemented by a mechanism preventing communication interruptions in the process. In this situation, the previous AA will mark its IP address as free in order to be allocated to some other node later.

The detection of duplicated addresses is undertaken in the PDAD process. Once any conflict has been detected, the AA which assigned the conflictive address is informed. This AA node will then generate a new *agentID* and it will warn all its dependant nodes to change their address from *agented + hostID* type to the new agentID.

With regards to the partitioning and merging of networks, the same above mentioned mechanism is utilized. In case of partitioning, the nodes AA will mark addresses which have left the network as free. In the case of the merging of two networks, the mechanism for the detection of duplicate addresses continues working properly.

#### 4.2.3. AROD

In *Address auto-configuration with address Reservation and Optimistic duplicated address Detection* (AROD) [24], the address reservation is based on the existence of nodes that have an IP address reserved to deliver it to the new nodes that enter. Two types of nodes will exist:

- Agents type 1 with a reserved IP address, apart from the IP address that has its network interfaces. When a node joins the network, this reserved IP will be assigned to it immediately.
- Agents type 2, which do not have reserved IP addresses. If a node that joins newly asks one of these for an IP address, this node borrows the reserved address of one of its neighbours who is of type 1, and it is assigned to the new one immediately.

It establishes a mechanism so that the network does not remain without nodes of type 1, whenever an IP address is assigned to a new node, the node that has delivered the address generates two random IP addresses (one for itself and another one for the new node that has joined) and does a DAD process to detect if these addresses are unique. Once the process has been carried out the following possibilities can arise:

- The IP addresses are unique; therefore, the two nodes turn into node type 1.
- If only one is unique, the one that gave the IP address will be turned into node type 1.
- If none is unique, the two nodes will remain as type 2.

This protocol considers its process of duplicated address detection as an optimistic DAD process, because it is only carried out once when a node joins and a reserved IP address is assigned to it. It contemplates the possibility of changing the reserved IP address number to the node type 1. If this number increases, the latency of IP address assignment is minor, but on the contrary the overhead increases due to having had to undergo more DAD processes, and *vice versa*. This possibility allows swapping latency *versus* overhead.

#### 4.2.4. AIPAC

*Automatic IP Address Configuration in Mobile* Ad Hoc *Networks* (AIPAC) [25] is a protocol for IP address auto-configuration using a reactive approach in the IP address assignment, so it should manage duplicate addresses; it is also focused on maintaining the address uniqueness after network merging due to the node mobility. The protocol has as its priority the supporting of the following features of *ad hoc* networks: limited resources of the devices and the unreliability in the wireless channels.

Each network is identified with its *NetID*. When two networks merge, and the merger is persistent, the *NetID* should be unified. To accomplish that, it uses a gradual fusion mechanism. This allows a node to pass from a *NetID* to another one, according to network changes observed by the node. This procedure allows a homogeneous system to be made in the case of multiple overlapping networks, according to the evolution of their topologies.

This protocol does not guarantee the uniqueness of the assigned IP addresses, but it ensures that messages are routed correctly. Each node in AIPAC is aware of its neighbouring radio, so the amount of information stored by the node is limited to the nodes within the radios distance.

### 4.3. Hybrid Protocols

#### 4.3.1. HCQA

*Hybrid Centralized Query-based Autoconfiguration* (HCQA) [26] was the first hybrid auto-configuration protocol. A node that wants to join the network undergoes a SDAD process. If the process is successful, the node will have to register its tentative IP address with an *Address Authority*. To do this, it will expect a message from the Address Authority and when that message has been received, it will send a registry request and the Address Authority will confirm it. The node starts a counter as this process begins, if the timer expires, it will start the process again until it can register the IP address. When the network is created, the first node becomes an Address Authority, it chooses a unique identifier for the network (e.g., MAC address) and advertises it periodically by *broadcast* messages to identify the network. If a node does not receive it, it is assumed that the network has been divided and it will create its own network becoming Address Authority. This protocol adds robustness to the SDAD process, it ensures no duplicity of IP addresses and it also provides a good mechanism for network *partitioning*. However, it has two main problems, firstly the overhead produced by the SDAD process and periodic messages of Address Authority, and secondly, the network depends on a central entity with which all nodes must communicate directly in order to register its IP address, so that much latency is added at the joining of nodes to the network.

#### 4.3.2. PACMAN

*Passive Autoconfiguration for Mobile* ad hoc *Networks* (PACMAN) [27] is a passive auto-configuration protocol for MANET. It uses elements from stateless and stateful protocols, so it could be considered somewhat hybrid. Its operation is based on each node assigning itself an address when joining the network, and passive monitoring of communications for the duplicate address detection.

To achieve the minimum overhead in the communications, the information is shared among different network layers. Specifically, the information handled by the routing protocol is monitored. The method used to choose the own IP address consists of a probabilistic algorithm. The probability of attempting to choose an IP address currently in use by another node is close to zero, this algorithm takes into account, among other factors, an assignment table. This table is created with information from the routing protocol on IP addresses already in use.

PACMAN uses the PDAD process to monitor the communications in search of duplicated addresses. This is necessary because the mechanism used for address assignment does not guarantee uniqueness (even if it attempts to reduce the probability of collision), and it may cause merging of networks containing nodes with the same IP address. Broadly speaking there are two types of events that indicate duplicity of IP addresses: firstly, we have the events that never occur if the address is unique, and always occur if the address is duplicated. These events confirm that there is a problem detected. On the other hand, we have the events that occur rarely if the address is unique, and often if the address is duplicated. In this way the possibility of problems is detected, so it is probabilistic algorithms. When it detects that two nodes are using the same IP address, it reports the problem to one of them using a *unicast* message to change its address. Moreover, it takes into account the problem of changing an address that has some communication going on. To fix this, when changing an address, a node notifies the nodes with which it has ongoing communications of its new IP address, so that they can make an encapsulation of the messages properly.

## 5. Performance Evaluation of Auto-Configuration Protocols

### 5.1. Performance Metrics for the Evaluation of Auto-Configuration Protocols

Zhou *et al.* [28] define some parameters that can be used to analyze the performance of an auto-configuration protocol for *ad hoc* networks (see Table 1).

### 5.2. Performance Evaluation of Studied Protocols

The submitted protocols share some common characteristics. However, they also differ in a wide range of issues. Table 2 presents a comparison of the characteristics of IPv4 addressing protocols.

## 6. Conclusions

The nodes of a network need a mechanism to exchange messages. The TCP/IP protocols can allow the different nodes of the same network to be associated with a different IP address. Due to the dynamic topology of mobile *ad hoc* networks (constant movement of the nodes that can enter and leave the network frequently or even simultaneously), auto-configuration protocols face many problems with guaranteeing the uniqueness of IP addresses. This work presents a classification of auto-configuration protocols for the most representative mobile *ad hoc* networks in literature with special emphasis on D2HCP protocol, and in particular in its design considerations and the advantages over their predecessors, especially when efficiently managing the IP address space of the *ad hoc* wireless network.

## Figures and Tables

**Table 1.** Evaluation metrics.

**Metrics**	**Description**
Uniqueness	Each MANET node must have a unique IP address for each network interface because duplicate addresses can cause serious routing problems.
Overhead	Exchanged packet number to obtain an IP address.
Latency	Node timeout to obtain the IP address.
Routing Protocol Independence	Auto-configuration protocols can work in two ways: leaning on a routing protocol to allow the routing of the new nodes joining the network, or regardless of routing algorithm.
Uniformity	All nodes perform the same function in the auto-configuration process.

**Table 2.** Comparison of the characteristics of IPv4 addressing protocols.

	**Protocol**	**Guarantee of Uniqueness**	**Overhead**	**Latency**	**Dependent Routing**	**Uniform**
**Stateful**	ManetConf	No	High	High	No	Yes
	DAAP	Yes	Medium	Medium	No	No
	Buddy System	Yes	Medium	Medium	No	Yes
	EMAP	No	Low	High	No	Yes
	D2HCP	Yes	Low	Low	Yes	Yes
**Stateless**	SDAD	No	High	High	No	Yes
	WDAD	No	Medium	Low	No	No
	PDAD	No	Medium	Low	No	Yes
	APAC	No	High	High	Yes	No
	AROD	Yes	High	High	No	No
	AIPAC	No	High	High	No	No
**Hybrid**	HCQA	Yes	High	High	Yes	No
	PACMAN	No	High	High	Yes	Yes
